# Bisphenols and Phthalates in Bottled Mineral Water: First Evidence of Co‐Occurrence, Estrogenic Activity, and Health Risk in Brazil

**DOI:** 10.1002/tox.70063

**Published:** 2026-02-23

**Authors:** Giulia Boito Reyes, Lucas Correia Gonçalves, Fernanda Cristina Iba Molinari, José Eduardo Gonçalves

**Affiliations:** ^1^ Programa de Pós‐Graduação Em Tecnologias Limpas (PPGTL) Universidade Cesumar (Unicesumar) Maringá Brazil; ^2^ Instituto Cesumar de Ciência, Tecnologia e Inovação (ICETI) Maringá Brazil; ^3^ Departamento de Farmácia (DFA) Universidade Estadual de Maringá (UEM) Maringá Brazil; ^4^ Departamento de Biomecidina Universidade Cesumar (Unicesumar) Maringá Brazil

**Keywords:** bisphenol, estrogenic activity, food contamination, phthalates, plastics

## Abstract

Bisphenol A (BPA), its structural analogs, and phthalates are synthetic chemicals widely used, with documented endocrine‐disrupting activity. This study aimed to develop and validate a selective multiresidue analytical method for the simultaneous determination of BPA, 10 bisphenol analogs, and six phthalates in PET‐bottled mineral water. To our knowledge, this is the first report assessing the co‐occurrence of these contaminants in bottled mineral water in Brazil. Six commercial bottled water brands were sampled under two storage conditions: ambient temperature and solar exposure. Solid‐phase extraction (SPE) was applied before GC–MS analysis. Among the 17 target analytes, BPA, BPF, BPS, and DIOP were detected in quantifiable concentrations. BPF and DIOP were found in all samples, with maximum concentrations of 7.92 and 3.85 μg L^−1^, respectively. BPA and BPS were detected in specific brands only after sunlight exposure, reaching up to 7.10 and 9.08 μg L^−1^, respectively. Despite concentrations being below current international regulatory limits, health risk assessment revealed that the estimated daily intake (EDI) of BPF and BPS resulted in safety factors (SF) below 1 for both adults and children, indicating a potential health concern. Estrogen equivalency (EEQ) values associated with BPA, BPF, BPS, and DIOP ranged from 0.5 to 13 ng E₂/L, exceeding effect‐based trigger values proposed for estrogenic activity in bottled mineral water. The results suggest that even BPA‐free labeled packaging may pose a risk due to the presence of its analogs and highlight the need for expanded regulatory oversight and routine monitoring of endocrine‐disrupting compounds in bottled water.

## 1 Introduction

With the advancement of industrialization, there has been a significant expansion in the global production chains of plastic materials. Currently, most manufactured plastics are non‐biodegradable, leading to continuous and persistent environmental pollution [1]. The situation becomes increasingly critical as plastic waste undergoes progressive fragmentation due to environmental conditions, resulting in the formation of microplastics (ranging from 0.1 to 5 mm in diameter) and nanoplastics (smaller than 0.1 μm). These particles exhibit high dispersal potential through both aquatic and atmospheric pathways [2].

Plastic polymers act as vectors for harmful chemical contaminants and pathogenic microorganisms. This occurs through a sorption process within the so‐called “plastisphere,” which refers to the interface between plastic surfaces and environmental microorganisms or biofilms. This phenomenon underscores the potential health and ecological threats arising from plastic degradation and contaminant leaching [3].

Due to their remarkable physical and chemical properties, bisphenol A (BPA) and its analogs are extensively used as components in plastic materials across a wide range of applications. These compounds are employed in manufacturing baby bottles, toys, household utensils, reusable water containers, beverage packaging (such as beer and soft drinks), and infant food containers. Moreover, they are also utilized in the pharmaceutical field, particularly in resins for medical and dental implants [4, 5].

Minimal concentrations of BPA in the human body can lead to a range of health issues, including infertility, endocrine disruption, and adverse effects associated with diabetes, cancer, obesity, and cardiovascular diseases [4, 6, 7]. Assessing the extent of contamination by organic residues resulting from plastic degradation, such as BPA and its analogs, remains a significant challenge, particularly in the extraction of these compounds from water samples.

Human exposure to BPA is widespread globally, primarily through food‐packaging materials. Among synthetic chemicals, BPA ranks among the highest in terms of global production volume. Although it has a relatively short shelf life, BPA is considered one of the most prevalent chemicals in the environment as it is constantly being released. Numerous studies have reported the migration of BPA from these coatings into various food items [8, 9, 10, 11]. Additionally, BPA is utilized in the manufacture of polycarbonate plastics found in items such as water bottles, optical discs, and dental sealants. Although polyethylene terephthalate (PET) is the predominant material used for producing bottled water containers and does not inherently require BPA in its polymerization process, traces of the compound have nonetheless been detected in PET‐bottled water samples [11, 12].

The detection of BPA in bottled mineral water and its associated adverse health effects [13, 14, 15] has prompted growing regulatory concern regarding its use in plastic packaging. In response, a variety of BPA analogs have been developed for similar applications [16]. These analogs (bisphenol S (BPS), bisphenol F (BPF), bisphenol AF (BPAF), bisphenol E (BPE), bisphenol B (BPB), bisphenol AP (BPAP), bisphenol Z (BPZ), bisphenol P (BPP)) have been identified in a range of environmental and biological samples [17, 18, 19, 20]. However, due to the structural similarity between these compounds and BPA, their safety for human health has been questioned [13, 14, 21]. Therefore, evaluating the presence of BPA and its analogs in bottled water is essential, particularly given that many products are labeled “BPA‐free,” yet systematic validation of such claims remains limited.

In response to increasing concerns about human exposure to BPA and its structural analogs through the consumption of bottled mineral water, this study was designed to develop a selective and reliable analytical method for their detection in bottled mineral water. The proposed approach integrates solid‐phase extraction (SPE) for sample pre‐concentration and cleanup, followed by sensitive identification and quantification using gas chromatography and mass spectrometry (GC–MS) to ensure robust detection and measurement.

## 2 Materials and Methods

### 2.1 Sample

For the analytical procedures, six commercial bottled mineral water brands packaged in 1.5 L PET containers were selected. The samples were purchased from local supermarkets in Maringá, Paraná, Brazil, on three different dates (01 April 2024, 27 June 2024, and 28 June 2024), capturing two distinct seasonal periods to increase representativeness. Among the selected brands, three were explicitly labeled as “BPA‐free.”

For each brand, three individual bottles (*n* = 3) from the same production batch were selected to ensure analytical consistency. Each bottle was treated as an independent sample and processed separately throughout the extraction, derivatization, and instrumental analysis steps. All analytical determinations (GC–MS runs) were performed in triplicate. The samples were stored under controlled laboratory conditions (25°C, protected from light) until the beginning of the experiments.

To simulate different storage conditions, the samples were subjected to two exposure scenarios: (i) room temperature (25°C), representing appropriate storage and transport conditions, and (ii) direct sunlight exposure, simulating inadequate storage and transportation environments. For the sunlight exposure treatment, the sealed PET bottles were placed under full direct sunlight for 8 h per day over 3 consecutive days (24 cumulative hours). Variations in temperature and global radiation are shown in Table S1. To ensure methodological reproducibility, the bottles were kept upright, with no contact with reflective surfaces. A control set of bottles was maintained simultaneously under controlled laboratory conditions (25°C ± 2°C, protected from light) to serve as baseline.

### 2.2 Reagents, Solvents and Standard Solutions

All reagents and solvents used in this study were of analytical or spectrophotometric grade. Ultrapure water, employed in the preparation of all aqueous solutions, was obtained from a Sartorius Arium Mini Ultrapure Water System.

Stock solutions (102 mg L^−1^) of each bisphenol standard—BPA, BPB, bisphenol C (BPC), BPE, BPF, bisphenol Fbis (BPFbis), BPS, BPZ, BPAF, BPAP, bisphenol A diglycidyl ether (BADGE)—and stock solutions (400 mg/L) of each phthalate standard—Diisooctyl phthalate (DIOP), Diisobutyl phthalate (DIBP), Dibutyl phthalate (DBP), Butyl benzyl phthalate (BBP), Di‐n‐octyl phthalate (DNOP), and Di(2‐ethylhexyl) phthalate (DEHP)—were prepared in methanol. All the standards were obtained from Sigma‐Aldrich (≥ 98% purity). Working solutions at a concentration of 1.0 mg L^−1^ were prepared in 10% methanol and stored in amber glass vials at 4°C. Calibration and analytical solutions were freshly prepared by appropriate dilution of the working solutions to obtain concentrations ranging from 1, 5, 10, 20, 50, 75, 100, and 150 μg L^−1^ for GC–MS analysis.

Control assays included (i) a blank sample (consisting of ultrapure water free of bisphenols and other reagents used in the analytical procedure), analyzed for the detection of traces of bisphenols and phthalates to rule out matrix interferences; and (ii) procedural blanks, in which the entire extraction process was carried out without the introduction of any sample, ensuring no contamination or analytical artifacts occurred during the bisphenols and phthalates determination.

### 2.3 Solid‐Phase Extraction (SPE) and Derivatization for GC–MS Analysis

For the extraction of bisphenol and phthalate residues, 1.5 L of bottled water samples were extracted in an SPE cartridge (Bond Elut ABS Nexus, 60 mg/3 mL, Agilent Technologies) at a constant flow rate of 1.0 mL min^−1^. After sample percolation, the cartridges were dried under a vacuum for 20 min to remove residual moisture. The adsorbed analytes were eluted sequentially with 3 mL of ethyl acetate, followed by 3 mL of methanol, with an enrichment factor of 250.

In the derivatization of bisphenol, 100 μL of dimethylformamide (DMF) and 100 μL of N,O‐bis(trimethylsilyl)trifluoroacetamide (BSTFA) containing 1% trimethylchlorosilane (TMCS) were added to 3 mL of the eluate. The mixture was vortexed for 30 s and then incubated at 60°C for 15 min. After derivatization, the samples were dried under a gentle stream of nitrogen and reconstituted in 1.0 mL of ethyl acetate for subsequent analysis by gas chromatography–mass spectrometry (GC–MS).

### 2.4 GC–MS Conditions

Chromatographic analyses were performed using an Agilent 7890B gas chromatograph coupled to an Agilent 5977A mass spectrometer detector (MSD) and equipped with an automatic sampler (CTC PAL Control). Separation of analytes was achieved on an HP‐5MS UI capillary column (Agilent Technologies).

The optimized oven temperature program was as follows: initial temperature of 100°C held for 2 min, ramped at 10°C min^−1^ to 280°C (held for 3 min), followed by a second ramp at 20°C min^−1^ to 310°C, with a final hold of 10 min. The injection volume was 1.0 μL in splitless mode. Helium was used as the carrier gas at a constant flow rate of 1.0 mL min^−1^.

The mass spectrometer was operated in electron impact (EI) mode at 70 eV. The ion source temperature was set at 230°C, quadrupole at 150°C, transfer line at 280°C, and injector at 250°C. Data acquisition was performed using the MassHunter Workstation software (Agilent Technologies).

Compound identification was based on retention times and mass spectra by comparison with analytical standards and the NIST 11 mass spectral library. Quantification was carried out in selected ion monitoring (SIM) mode using characteristic retention times and quantifier ions for each bisphenol compound [22].

### 2.5 Risk Assessment

#### 2.5.1 Daily Intake‐Associated Risk Assessment

The estimated human daily intake (EHDI) for each bisphenol and phthalate analog detected in bottled mineral water was calculated following the methodology described by Wang [23] and Luo [22], using the equation:

(1) $$\text{EHDI}=\frac{\mathrm{Ci}\times V}{W}$$

where EHDI represents the estimated human daily intake (μg/kg/day), *Ci* is the concentration of each bisphenol and phthalate analog in bottled mineral water (μg/L), *V* is the daily water intake (L/day), and *W* is the average body weight. For this study, the assumed body weights were 70 kg for adults (> 18 years) and 12 kg for children (3 years), with corresponding daily water consumption values of 2.25 L (man 2.5 L and woman 2.0 L) and 1.3 L, respectively, as recommended for the Brazilian population [22, 23, 24, 25].

To assess potential health risks, the safety factor (SF) was calculated as the ratio between the established reference dose (RfD) for each bisphenol and phthalate analog and the corresponding estimated daily intake (EHDI), according to Wang [23] and Luo [22]:

(2) $$\mathrm{SF}=\frac{\mathrm{RfD}}{\text{EHDI}}$$

#### 2.5.2 Estrogenic Activity‐Associated Risk Evaluation

To evaluate the cumulative estrogenic activity associated with bisphenol and phthalate analogs detected in bottled mineral water, the Estrogenic Equivalency (EEQ) was calculated for each compound based on the approach proposed by Wang [23] and Luo [22]. The EEQ provides an estimate of the potential endocrine‐disrupting activity of a mixture of compounds, normalized to the estrogenic potency of a reference compound (typically 17β‐estradiol),

(3) $$\mathrm{EEQ}=\sum \left(\mathrm{EPi}\times \mathrm{Ci}\right)$$

where EPi is the estrogenic potency of the *i*‐th bisphenol analog, expressed as a fraction relative to the potency of 17β‐estradiol, and *Ci* is the corresponding concentration (μg/L) of the compound in the bottled water samples.

This approach enables the comparison of the combined estrogenic activity of structurally related endocrine‐disrupting chemicals, taking into account both their concentration and their relative biological potency. The EEQ values obtained can be used to estimate the potential endocrine risk posed by chronic exposure to bisphenol analogs via drinking water consumption.

## 3 Results and Discussion

Variations in the manufacturing conditions of commercial plastic bottles can significantly influence the release of bisphenols and phthalates into the contents of the container [10, 11]. Bisphenols, in particular, may be incorporated during the plastic expansion process, potentially remaining as residual monomers or by‐products [26]. Consequently, the presence of bisphenol residues in bottled water may be attributed to several factors, including the production technology, bottling processes, cross‐contamination during packaging, and the chemical quality or purity of the raw materials used [27]. These findings align with recent studies reporting that thermal conditions, polymer formulation, and mechanical stresses during bottling can facilitate the leaching of endocrine‐disrupting compounds (EDCs) into beverages, even under normal storage conditions [28, 29].

In Brazil, PET bottles are commonly used for bottling mineral water due to their favorable properties, including limited additives and a relatively low diffusion rate of harmful organic compounds. However, factors such as background contamination, the source of the water, pre‐bottling processes, and the recycling of PET can contribute to the presence of bisphenols and phthalates in bottled water. Studies have shown that recycled PET (rPET) may contain higher levels of contaminants due to multiple sources of contamination and variability in the recycling process, potentially leading to increased migration of substances like BPA into bottled water [30, 31, 32].

Recent investigations have also raised concerns about “BPA‐free” plastic bottles, which are marketed as safer alternatives. Research indicates that these bottles may still release EDCs such as BPS and BPF under certain conditions, including exposure to heat and light. This underscores the need for comprehensive assessments of all materials used in food and beverage packaging, regardless of their BPA content. This highlights the need for rigorous regulatory oversight and standardized testing protocols to verify the chemical safety of BPA‐free labeled materials, especially when used for consumables [10].

For the determination of bisphenols and phthalates in PET‐bottled mineral water, an analytical method adapted from Wang [23] was employed. The methodology involved solid‐phase extraction (SPE) followed by gas chromatography coupled with mass spectrometry (GC–MS). The adapted method was fully optimized based on the detection frequency observed in the analyzed samples, ensuring selectivity, linearity, accuracy, precision, limits of detection (LOD), limits of quantification (LOQ), and matrix effects in accordance with the criteria established by the United States Environmental Protection Agency (USEPA) [33].

Matrix effects were assessed using ultrapurified water as a blank matrix. The validation process confirmed the method's robustness and suitability for trace‐level quantification of target analytes in complex aqueous matrices. A comprehensive summary of the method validation parameters is presented in Table 1.

**TABLE 1:** Validation parameters of the extraction and chromatographic analysis method for bisphenols and phthalates in bottled mineral water.

Compounds	LOD (μg L^−1^)	LOQ (μg L^−1^)	REC (%)	*R* ^2^
BPA	0.56	1.57	96.5	0.9970
BPB	0.49	1.85	97.9	0.9965
BPC	0.88	1.03	91.7	0.9911
BPE	0.65	1.05	96.2	0.9972
BPF	0.91	2.99	93.5	0.9976
BPS	2.05	3.49	93.6	0.9908
BPZ	1.97	3.21	92.2	0.9938
BPFbis	0.77	1.15	95.5	0.9934
BPAF	0.99	2.41	90.4	0.9875
BPAP	0.59	1.02	96.6	0.9947
BADGE	1.99	4.05	90.3	0.9876
DIOP	2.14	4.13	92.8	0.9889
DIBP	1.39	2.29	94.4	0.9908
DBP	1.18	2.09	92.6	0.9956
BBP	1.96	3.57	91.1	0.9913
DNOP	2.34	4.06	90.7	0.9779
DEHP	2.27	4.12	90.5	0.9930

Abbreviations: BADGE = Bisphenol A diglycidyl ether; BBP = Butyl benzyl phthalate; BPA = Bisphenol A; BPAF = Bisphenol AF; BPAP = Bisphenol AP; BPB = Bisphenol B; BPC = Bisphenol C; BPE = Bisphenol E; BPF = Bisphenol F; BPFbis = Bisphenol Fbis; BPS = Bisphenol S; BPZ = Bisphenol Z; DBP = Dibutyl phthalate; DEHP = Di(2‐ethylhexyl) phthalate; DIBP = Diisobutyl phthalate; DIOP = diisooctyl phthalate; DNOP = Di‐n‐octyl phthalate; LOD = Limit of detection; LOQ = Limit of quantification; *R* ^2^ = coefficient of determination; REC = recovery.

The adapted method demonstrated excellent analytical performance, with determination coefficients (R [2]) exceeding 0.99 for BPA, BPF, and BPS, and greater than 0.98 for DIOP. Recovery rates were consistently above 92%, while limits of detection (LOD) and quantification (LOQ) ranged from 0.56 to 2.14 μg L^−1^ and 1.57 to 4.13 μg L^−1^, respectively (Table 1). These results indicate that the optimized method for simultaneous analysis of phthalates and bisphenols in bottled mineral water exhibits analytical characteristics comparable to those reported in previous studies focused on phthalates [22, 34] and bisphenols [12, 22, 35, 36] in bottled water samples.

### 3.1 Phthalate and Bisphenol Analysis in Bottled Mineral Water Samples

Concentrations of six phthalates and eleven bisphenols were investigated in six brands of bottled mineral water packaged in PET bottles from the southern region of Brazil. As shown in Table 2, only one phthalate (DIOP) and three bisphenols (BPA, BPF, and BPS) were detected, all within the maximum allowable limits established by legislation [38]. The bisphenol analogs BPB, BPC, BPE, BPZ, BPAF, BPAP, BADGE, and BPFBIS were not detected in any of the samples analyzed.

**TABLE 2:** Mean concentrations (± SD) of bisphenols and phthalates detected in the six analyzed bottled water brands with standard deviation values.

Sample	TA/ES	BPA	BPF	BPS	DIOP
1	TA	nd	7.19 ± 0.54^A,a^	9.08 ± 0.29^d^	3.74 ± 0.09^C,c^
	ES	nd	6.44 ± 2.29^A,a^	nd	3.64 ± 0.15^C,c^
2	TA	nd	6.57 ± 0.09^A,a^	nd	3.80 ± 0.19^C,c^
	ES	nd	6.06 ± 1.87^A,a^	nd	3.61 ± 0.02^C,c^
3	TA	nd	6.45 ± 0.22^A,a^	nd	3.69 ± 0.15^C,c^
	ES	nd	7.92 ± 4.2^A,a^	nd	3.63 ± 0.06^C,c^
4	TA	nd	5.82 ± 0.28^B,b^	nd	3.85 ± 0.21^C,c^
	ES	nd	5.82 ± 1.75^B,b^	nd	3.62 ± 0.05^C,c^
5	TA	nd	5.75 ± 1.01^B,b^	nd	3.70 ± 0.08^C,c^
	ES	7.10 ± 0.21^e^	4.82 ± 0.11^B,b^	8.73 ± 0.33^e^	2.40 ± 0.13^C,c^
6	TA	nd	5.52 ± 0.09^B,b^	nd	3.66 ± 0.12^C,c^
	ES	nd	7.70 ± 3.76^A,a^	nd	3.68 ± 0.06^C,c^
MPL		50 μg/kg [37]	200 μg/kg [37]	50 μg/kg [37]	—

*Note:* The results represent the means ± standard deviation in triplicate. Different letters indicate significant differences by Tukey's test (≤ 0.05). Equal values with uppercase letters in columns (TA and ES), lowercase letters in lines (Bisphenol and Phthalate) do not differ significantly according to Tukey's test.

Abbreviations: BPA = Bisphenol A; BPF = Bisphenol F; BPS = Bisphenol S; DIOP = diisooctyl phthalate; ES = sun exposure; MPL = maximum permitted limit per kg of food; nd = not detected; TA = room temperature.

DIOP and BPF were found in all six PET bottled water brands, with mean concentrations ranging from 2.40 to 3.85 μg L^−1^ and 4.82 to 7.92 μg L^−1^, respectively. According to previous studies [9, 11, 20, 22], the migration of phthalates from plastic packaging into bottled water can be influenced by several environmental and storage‐related factors, including temperature, storage duration, sunlight exposure, and pH conditions, which affect the degradation and permeability of the polymer matrix rather than the analytical process itself. High temperatures and prolonged storage have been shown to increase the rate of plastic degradation and, consequently, the release of additives, while sunlight, particularly UV radiation, may further enhance polymer oxidation and facilitate the diffusion of bisphenols into the aqueous phase [20, 22]. In the present study, no pH adjustment was performed before SPE extraction; samples were analyzed under their original physicochemical conditions to reflect realistic consumer exposure.

BPA was detected exclusively in sample 5 after 24 h of solar exposure at a concentration of 7.10 ± 0.29 μg L^−1^, indicating that elevated temperatures induced by sunlight may enhance BPA migration into the water. BPS was found in only two samples: 9.08 ± 0.29 μg L^−1^ in sample 1 under ambient temperature and 8.73 ± 0.33 μg L^−1^ in sample 5 after solar exposure. These findings align with those reported by Dybdahl [39], who demonstrated that bisphenol concentrations in bottled water vary according to exposure conditions (temperature fluctuations) and the specific bisphenol compound.

The detected levels, although below regulatory limits, underscore the need for continued monitoring given the potential endocrine‐disrupting effects of these contaminants, especially under varying storage and environmental conditions [40, 41].

To the best of our knowledge, and based on the current scientific literature, this is the first study to report the presence of BPF, BPS, and DIOP in bottled mineral water packaged in PET containers. Only a few studies have reported the detection of BPF (0.012–0.014 ng mL^−1^) and BPS (0.011–0.015 ng mL^−1^), and these were exclusively in urine samples from Thai school‐aged children [42], suggesting that the substitution of BPA by its analogs may still result in contamination of water and food intended for human consumption.

Wang et al. [23] reported the widespread presence of BPE and BPS in commercial bottled waters in China, suggesting that the replacement of BPA by structurally similar analogs has become common practice in polymer manufacturing. Luo et al. [22] further demonstrated that environmental stressors, including storage temperature, UV exposure, and physicochemical properties of the polymer matrix, can facilitate the migration of phthalates and bisphenols from PET packaging into drinking water. The detection of DIOP across all brands in our study aligns with these migration mechanisms, reinforcing the observation that phthalate esters continue to leach from PET materials despite regulatory restrictions.

Because it has been widely reported in the scientific literature [12, 23, 36] with numerous studies highlighting its high toxicity, BPA has been replaced by its analogs. This substitution allows packaging to be labeled as “BPA‐free.” Its most common analogs, although with little known toxicological effects, are BPF and BPS. However, recent studies have shown that the effects of BPF and BPS on human health are very similar to those of BPA [43, 44, 45, 46].

### 3.2 Health Risk Assessment and Estrogenic Activity Associated With Bisphenol and Phthalate Intake Through Bottled Mineral Water

Table 3 presents the estimated average daily intake values for adults and children based on the mean concentrations of bisphenols and phthalates detected in PET‐bottled mineral water, under both room temperature conditions and after solar exposure. Additionally, the table includes the associated estrogenic activity (EEQ) and safety factor (SF) values, enabling a comprehensive assessment of potential health risks related to endocrine‐disrupting activity.

**TABLE 3:** Estimated exposure of adults and children to bisphenols and phthalates through consumption of PET‐bottled mineral water.

Parameter	BPA	BPF		BPS		DIOP	
	ES	TA	ES	TA	ES	TA	ES
Mean concentration (μg L^−1^)	7.10	7.19	7.92	9.08	8.73	3.85	3.68
Risk assessment associated with daily intake (adult)	0.23	0.23	0.25	0.29	0.28	0.12	0.12
Risk assessment associated with daily intake (child)	0.77	0.78	0.86	0.98	0.94	0.42	0.39
Safety factor (adult)	219.1	0.001	0.001	0.027	0.028	161.61	169.1
Safety factor (child)	65.0	0.0004	0.0004	0.0082	0.0085	48.0	50.2
Estrogenic activity associated with risk assessment (ngE2/L)	13	5	6	0.5	0.5	3.8	3.7
Estrogenic potential	1.9 × 10^−3^ [47]	7.2 × 10^−4^ [47]		5.5 × 10^−5^ [47]		1 × 10^−3^ [48]	
Reference dose (RfD) (μg/kg/dia)	50 [49]	0.00037 [4]		0.00809 [4]		20 [50]	

Abbreviations: BPA = Bisphenol A; BPF = Bisphenol F; BPS = Bisphenol S; DIOP = diisooctyl phthalate; ES = sun exposure; TA = room temperature.

The health risk assessment associated with the estimated average daily intake of bisphenols and phthalates by adults and children was conducted based on the mean concentrations detected in PET‐bottled mineral water samples. Safety factors (SF) were calculated using established reference doses (RfD) for BPA and DIOP/DEHP [33, 38]. The resulting SF values were considerably greater than 1 for both population groups, indicating that the concentrations of BPA and DIOP detected in the samples do not pose a significant health concern under the evaluated exposure conditions. These findings are consistent with previous studies [12, 22, 23], which have similarly concluded that PET‐bottled mineral water generally poses a low risk to human health regarding contamination by these compounds.

In contrast, the calculated safety factors (SF) for BPF and BPS were significantly below 1 for both adults (BPF 0.001 for TA and ES; BPS 0.027 for TA and 0.028 for ES) and children (BPF 0.0004 for TA and ES; BPS 0.0082 for TA and 0.0083 for ES), indicating that the concentrations of these bisphenol analogs detected in PET‐bottled mineral water may pose a considerable health risk (SF < 1 indicates potential non‐compliance with safety margins [22, 23]). According to Cao [4], exposure to BPF and BPS has been associated with a decline in semen quality in men, including reductions in ejaculate volume and sperm count. These findings raise concerns regarding the endocrine‐disrupting potential of BPA substitutes and highlight the need for further toxicological evaluation of BPF and BPS, especially in vulnerable populations.

Correlating the findings of Cao et al. with the World Health Organization's reference values [51], exposure to BPF and BPS was associated with an increased proportion of men presenting below‐threshold values for sperm concentration, total sperm count, progressive motility, and overall motility. These alterations further support the hypothesis that, similar to BPA, both BPF and BPS exhibit endocrine‐disrupting activity and may contribute to male reproductive dysfunction.

In pregnant women, BPA has been shown to cross the placental barrier, leading to elevated intrauterine concentrations and direct fetal exposure [52]. Recent studies have also confirmed that BPS can be transferred to the fetus, with evidence suggesting higher BPS levels in fetal circulation than in maternal blood. Furthermore, BPS has been found to persist in the uterine cavity [53, 54], potentially impairing neurodevelopment at 2 years of age [55], and altering endocrine system function at ages 4 and 6 [56]. Regarding BPF, although its accumulation in amniotic fluid has been observed within 72 h of exposure, no conclusive evidence is yet available confirming its retention within the uterine environment [53].

In addition to developmental and reproductive toxicity, BPS exposure has been linked to hepatic dysfunction. Studies by Mornagui [57] demonstrated that BPS can induce hepatic hypertriglyceridemia, resulting in liver damage and histopathological alterations such as inflammatory cell infiltration, hepatocellular necrosis, and steatosis.

BPF exposure has also been linked to a range of adverse biological effects. These include disruptions in sperm physiology and spermatogenesis [58], interference with genital development [59], and impairment of cholesterol/testosterone homeostasis [58]. Furthermore, BPF inhibits fatty acid and lipid metabolism in the liver, leading to hepatic injury [29], and disrupts thyroid function by altering the regulation of thyroid hormone T4 [60]. BPF has also demonstrated potential neurotoxic and immunotoxic effects in humans.

Additional health concerns related to bisphenol and its analogs include an elevated risk of cardiovascular diseases, particularly among older adults and individuals with overweight or obesity. Other reported outcomes include endocrine disruption, reproductive disorders, and carcinogenesis [43, 44, 45]. Prolonged ingestion of bisphenols has also been associated with chronic diseases such as type II diabetes mellitus, obesity, congenital malformations, chronic respiratory and renal conditions, breast cancer, infertility, and spontaneous abortion [61]. These findings corroborate the growing concern that BPS and BPF, similar to BPA, pose significant health risks through endocrine disruption and metabolic disturbances.

Estrogenic activity values (EEQ), initially reported in [μgE₂/L], were converted to [ngE₂/L] to facilitate comparison with ecotoxicological benchmarks from previous studies. The EEQ concentrations detected in PET‐bottled mineral water samples ranged from 0.5 to 13 ngE₂/L, with an average value of 4.8 ngE₂/L. According to the effect‐based trigger values (EEQ) proposed by Neale [62], the recommended EEQ for estrogenic activity in drinking water is approximately 0.1 ngE₂/L. Concentrations exceeding this threshold indicate a potential concern for endocrine disruption and warrant further investigation through chemical confirmation and risk characterization.

Therefore, even the lowest EEQ concentration observed in this study (0.5 ngE_2_/L) exceeds the EEQ by a factor of five, suggesting that the estrogenic potential of bottled mineral water under the evaluated storage conditions is not negligible. The highest recorded value (13 ngE_2_/L) surpasses the EEQ by over two orders of magnitude, reinforcing the hypothesis that cumulative low‐level exposure to bisphenol analogs may represent an underrecognized source of endocrine‐disrupting activity in commercially available drinking water. These findings indicate that the estrogenic potential of bottled mineral water under current storage and handling conditions is not negligible and may contribute to low‐level, chronic exposure to EDCs, as advocated by Neale [62].

Collectively, these results reveal a concerning scenario consistent with prior studies reporting estrogenic activities linked to bisphenols and phthalates in drinking water. The data emphasize the need to integrate effect‐based monitoring into water safety evaluations and to establish stricter regulatory measures for estrogenic contaminants in bottled drinking water.

## 4 Conclusion

This study describes the development and validation of a robust and selective multiresidue analytical method for the determination of bisphenols and phthalates in PET‐bottled mineral water. Among the compounds analyzed, BPA, BPF, BPS, and DIOP were detected, with concentrations remaining below current regulatory limits. However, the estimated daily intake (EDI) and calculated safety factors (SF) for BPF and BPS, particularly in samples exposed to sunlight, revealed potential health concerns, especially for vulnerable groups such as children.

Although BPA and DIOP did not present significant risk based on current reference doses, the detection of BPF and BPS at levels associated with endocrine‐disrupting potential raises serious concerns. These findings align with recent toxicological evidence suggesting that BPA analogs may exhibit similar or even more potent biological activity than BPA itself.

To our knowledge, this is the first report confirming the presence of BPF and BPS in PET‐bottled mineral water marketed in Brazil. Given the growing use of BPA substitutes in consumer plastics, these results underscore the urgent need to broaden the regulatory scope to include structurally related bisphenol analogs. Continuous monitoring of these contaminants in drinking water, especially under conditions simulating typical storage and transport scenarios, is essential to support future risk assessments and public health policies.

## Author Contributions

**Giulia Boito Reyes:** conceptualization, data curation, formal analysis, investigation, methodology, writing – review and editing. **Lucas Correia Gonçalves:** methodology, writing – review and editing. **Fernanda Cristina Iba Molinari:** data curation, formal analysis, methodology. **José Eduardo Gonçalves:** designed the study, conceptualization, investigation, methodology, writing – review and editing, supervision.

## Funding

The authors have nothing to report.

## Conflicts of Interest

The authors declare no conflicts of interest.

## Supporting information


**Table S1:** Variations in temperature (°C) and radiation (kJ/m^2^) on the days of solar exposure [63].

## Data Availability

The data that support the findings of this study are available on request from the corresponding author. The data are not publicly available due to privacy or ethical restrictions.
