# Supplementary material for: Bisphenols and Phthalates in Bottled Mineral Water: First Evidence of Co‐Occurrence, Estrogenic Activity, and Health Risk in Brazil
Source: Environ Toxicol. 2026 Feb 23;41(11):1179–87. doi: 10.1002/tox.70063 (PMC13656891; doi:10.1002/tox.70063)
Supplement: Supplementary file 1 [file TOX-41-1179-s001.docx]

**Supplementary Material**

**Bisphenols and Phthalates in** **Bottled Mineral Water: First Evidence of Co-Occurrence, Estrogenic Activity, and Health Risk in Brazil**

Giulia Boito Reyes^1,4^, Lucas Correia Gonçalves^2^, Fernanda Cristina Iba Molinari^3^, José Eduardo Gonçalves^1,4^

^1^Programa de Pós-Graduação em Tecnologias Limpas (PPGTL), Universidade Cesumar (Unicesumar), Maringá, Brazil.

^2^Departamento de Farmácia (DFA), Universidade Estadual de Maringá (UEM). Maringá, Brazil,

^3^Departamento de Biomecidina, Universidade Cesumar (Unicesumar), Maringá, PR, Brazil.

^4^Instituto Cesumar de Ciência, Tecnologia e Inovação (ICETI), Maringá, PR, Brazil.

*Corresponding author: Universidade Cesumar (Unicesumar) and Instituto Cesumar de Ciência, Tecnologia e Inovação (ICETI), Brazil; e-mail address: jose.goncalves@unicesumar.edu.br.

Table S1. Variations in temperature (°C) and radiation (kJ/m²) on the days of solar exposure^63^.

| **Days of solar exposure** | T_Max_ **(°C)** | T_Min_ **(°C)** | R_Max_**(kJ/m²)** | R_Min_**(kJ/m²)** |
| --- | --- | --- | --- | --- |
| April 29, 2024 | 32,8 | 23,1 | 2940,4 | 273,4 |
| April 30, 2024 | 33 | 23 | 2884,6 | 158,4 |
| May 1, 2024 | 33 | 23 | 2878,6 | 233,8 |
| June 28, 2024 | 27,5 | 15,2 | 1789,4 | 80,7 |
| June 29, 2024 | 23,9 | 18,8 | 2125,9 | 41,6 |
| June 30, 2024 | 22,7 | 10,6 | 2162,2 | 85,1^4^ |

T_Max_ = Maximum Temperature; T_Min_ = Minimum Temperature; R_Max_ = Maximum Global radiation; R_Min_ = Minimum Global radiation.

REFERENCE:

INM. Instituto Nacional de Meteorologia: Dados Históricos Anuais, [Instituto Nacional de Meteorologia - INMET](https://portal.inmet.gov.br/dadoshistoricos#:~:text=Instituto%20Nacional%20de%20Meteorologia%20%2D%20INMET)
